# Breeding fat‐tailed dunnarts (*Sminthopsis crassicaudata*) in captivity: Revised practices to minimize stress whilst maintaining considerations of wild biology

**DOI:** 10.1002/dvdy.755

**Published:** 2025-02-02

**Authors:** Emily L. Scicluna, Axel H. Newton, Jennifer C. Hutchison, Alicia M. Dimovski, Kerry V. Fanson, Gail D'Souza, Shiralee Whitehead, Andrew J. Pask

**Affiliations:** ^1^ TIGRR Lab, The School of BioSciences University of Melbourne Parkville VIC Australia; ^2^ Department of Environment and Genetics Research Centre for Future Landscapes School of Life Sciences La Trobe University Bundoora VIC Australia; ^3^ Centre for Reproductive Health Hudson Institute of Medical Research Clayton VIC Australia; ^4^ Department of Molecular and Translational Science Monash University Clayton VIC Australia; ^5^ Department of Animal, Plant and Soil Sciences, School of Agriculture, Biomedicine and Environment La Trobe University Bundoora VIC Australia

**Keywords:** captive breeding, endocrinology, glucocorticoids, hormones, husbandry, mammal, marsupial, stress

## Abstract

**Background:**

The fat‐tailed dunnart is a small dasyurid marsupial which is emerging as a robust laboratory model for conservation, developmental, and reproductive biology research. While these marsupials present extremely valuable models, housing non‐domesticated animals in captivity can present a wide range of potential stressors for the animals, which need to be managed to ensure colony health. Notably, dunnarts rely on scent marking for social communication, which is important to maintain to reduce stress in artificial environments.

**Results:**

In this study, we examine captive management techniques and provide updated recommendations which consider both scientific and conservation outcomes. Through ongoing management, we observe that recapitulating aspects of a natural environment has a significant impact on stress reduction and improving the overall reproductive fitness of captive‐bred colonies. Moreover, our study provides evidence for preferred cage base substrate types, and quantification of stress caused by the cadence of enclosure cleaning using fecal glucocorticoid metabolite levels as an indicator of stress.

**Conclusion:**

The study underscores the significance of population management in captive breeding programs, advocating for maintaining genetic diversity and meticulous record‐keeping. We have further refined best practice for managing captively bred dunnart colonies, outlining guidelines for enclosure requirements, handling, cleaning, feeding, and lighting during breeding. Overall, the research aims to improve the health and productivity of captive fat‐tailed dunnarts, ensuring their continued contribution as a valuable laboratory‐based marsupial model and aiding in the conservation of related endangered species, while meeting a balance between maintenance of strict hygiene and alignment with wild‐life history.

## Key Findings

- This study provides detailed guidelines for managing captively bred dunnart colonies. Best practices for enclosure requirements, handling, cleaning and feeding. Detail methods to reduce stressors in captive animals. Detailed processes for husbandry, breeding and raising pouch young.

## 1 INTRODUCTION

Fat‐tailed dunnarts (*Sminthopsis crassicaudata*) are a small, carnivorous marsupial of the family Dasyuridae, order Dasyuromorphia. This species is nocturnal and is found in a range of habitats in the wild, which are widespread across Australia.^1^ Captive breeding colonies of fat‐tailed dunnarts have been established across Australia since the 1960s,^2^, ^3^, ^4^ and some of these have been actively running since then. Dunnarts make for an ideal model species for marsupial biological and conservation research, owing to their small size, short gestation, polyovulatory nature, and the ability for their natural reproductive cycles to be manipulated for year‐round breeding. As such, dunnarts have served as a valuable model for reproduction and embryology,^4^, ^5^, ^6^ developmental biology,^7^ genetics, and genomics.^8^

To ensure accurate scientific insights and promote optimal conservation outcomes, it is important to use the best possible husbandry practices. For their continued use as a laboratory marsupial model,^5^ assessing individual levels of acclimation to captivity is integral if long‐term goals are aligned with wild comparability, or even wild compatibility and survival. This is particularly pertinent to conservation efforts as many Dasyurids are the focal point of national recovery programs, including but not limited to the Kangaroo Island dunnart (*Sminthopsis aitkeni*), Julia‐creek dunnart (*Sminthopsis douglasi*), sandhill dunnart (*Sminthopsis psammophilia*), Tasmanian devil (*Sarcophilus harrisii*), northern quoll (*Dasyurus hallucatus*), eastern quoll (*Dasyurus viverrinus*), and numbat (*Myrmecobius fasciatus*). The fat‐tailed dunnart has recently been listed as threatened in Victoria and included in the Flora and Fauna Guarantee Act 1988,^9^ making new conservation strategies for this species of high importance. Lessons learned from the fat‐tailed dunnarts are integral to the conservation of not only their own threatened species but may also be transferrable to their more endangered relatives.

While dunnarts have been successfully bred in captivity for decades, there are conflicting reports on best practices for the long‐term maintenance of this species. In this resource article, we review previous standards for establishing and maintaining a fat‐tailed dunnart laboratory colony, and provide perspectives into updated research and standardized procedures to increase the health, productivity and wellbeing of captive‐bred animals. We specifically investigate enclosure conditions as potential stressors, finding certain substrates and cleaning regimes increase fecal glucocorticoid metabolite (FGMs) levels and stress on animals. Furthermore, the best practices for animal wellbeing detailed in this study have been applied in a companion study to successfully establish methods for husbandry and timed embryo collection to examine dunnart embryonic development.^5^ Through application of these new and improved methods of animal husbandry and monitoring with close consideration of wild biology, this species can serve as a valuable marsupial model for both research and conservation.

## 2 RESULTS AND DISCUSSION

The observations and perspectives outlined in this study were established within a captive laboratory colony of fat‐tailed dunnarts at the University of Melbourne, with incorporated learnings from the varying management practices of past colonies. Captive fat‐tailed dunnarts are often housed in laboratory‐type, precisely managed environments for use in scientific experimentation. Long‐term housing in a controlled, invariable environment can lead to morphological, behavioral, cognitive and genetic change as animals become accustomed to predictability, differentiating individuals from their wild counterparts.^10^ Key life history traits (Tables 1 and 2) should be considered when managing a captive colony, and active management can ensure acclimation to captivity is minimized.

**TABLE 1:** Summary of fat‐tailed dunnart life history and morphological traits.

Biological trait	Measurement	Reference
Head and body length (mm)	60–90 (mean 70)	Menkhorst and Knight, 2013^11^
Tail length (mm)	45–70 (mean 60)	Menkhorst and Knight, 2013^11^
Weight (g)	10–23 (mean 16)	Newton et al.^5^
Life expectancy (years)	Wild: Males 1–2	Morton, 1976^12^
	Females: 2–3	Morton, 1976^12^
	Captive: both sexes ≤5	E. Scicluna pers. obs.
Gestation (days)	13–16 (mean 14)	Godfrey and Crowcroft^2^; Newton et al.^5^
Oestrus cycle (days)	31.1 ± 0.7	Godfrey and Crowcroft^2^; Newton et al.^5^; Smith et al., 1978^13^
Litter size	~5–7, maximum 10	Newton et al.^5^; Morton^14^
	(nipple number = 10)	
Birth weight (mg)	~12	Cook et al.^7^
Wild breeding season	*July–February*	Morton^1^
	First litters of season:	Morton^14^
	July–October	
	Second litters of season:	
	November–February	

**TABLE 2:** Summary of key fat‐tailed dunnart postnatal reproductive and developmental stages.

Reproductive timeline (days–postnatal)	Growth stage	Reference
0	Birth weight 12 mg, Length size 3–5 mm	Cook et al.^7^
2	Female pouch young: pouch primordia visible, external genitalia visible (shallow pouch depression)	Cook et al.^7^
4	Male pouch young: scrotal bulge visible	Cook et al.^7^
37	Young protruding from mother's pouch	Godfrey and Crowcroft^2^; Ewer^3^
40	Pouch young fully covered in fur	Cook et al.^7^
41–50	Pouch young permanent attachment to nipples ceases; young can switch nipples or leave pouch temporarily. Sometimes carried on mother's back or left in nest	Ewer^3^; Suarez et al., 2017^15^
51–70	Pouch young permanently exit the pouch and commence eating solid food	Suarez et al., 2017^15^
59–63	Pouch young first start leaving nest	Ewer^3^
60	Pouch young eyes open. Male pouch young: testes descend into scrotum	Cook et al.^7^
65–70	Pouch young weaning from mother; body weight 5–8 g	Godfrey and Crowcroft^2^; Bennett et al.^16^
85 (~3 months)	Females sexually mature (1st oestrous)	Bennett et al.^16^
200 (~7 months)	Males sexually mature	Bennett et al.^16^
37 months (3 years)	Maximum breeding age for females	La Trobe University^17^; University of Melbourne^18^

### 2.1 Housing conditions

Fat‐tailed dunnarts have been housed and bred successfully in a variety of enclosure types in a laboratory setting, with a minimum recommended floor space of 50 × 35 × 30 cm.^2^, ^16^, ^17^, ^19^, ^20^ To encourage maintenance of “wild” behaviors, enclosures as large as possible are recommended, to reduce stress and therefore the influx of stereotypic behaviors due to a lack of adequate stimulation. Larger enclosures and adequate hiding spaces will allow the closer representation of wild behavior, including the establishment of territories, and enrichment through an increased ability to navigate surroundings. Enclosure height should allow vertical space for jumping, and be at least 40 cm tall to avoid escape when the enclosure roof is removed.

**FIGURE 1:**
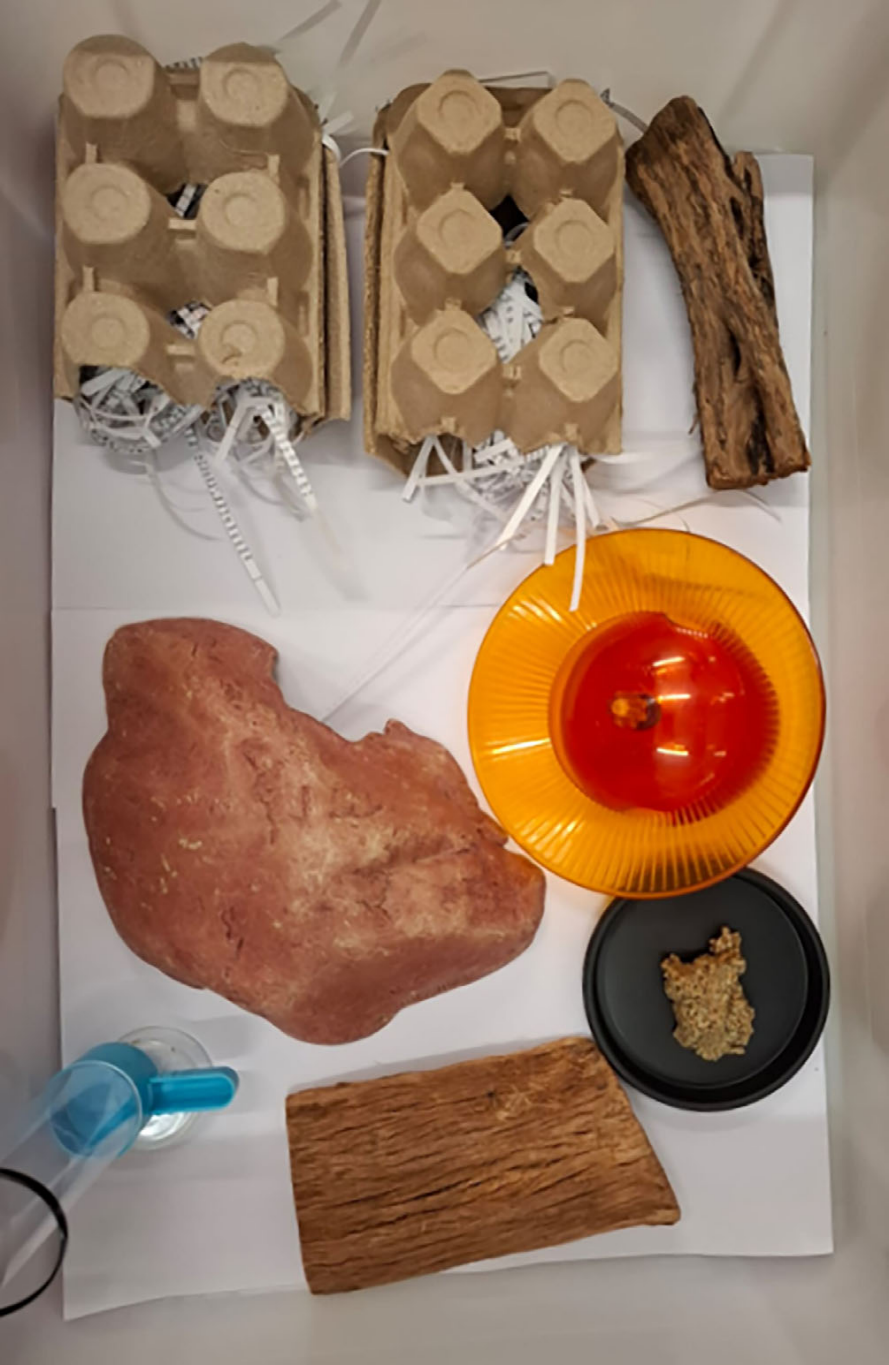
Diagram of enclosure setup containing four shelter sites (two egg‐cartons, one “rock” hide, and one bark tunnel) with extra bark piece and running wheel for enrichment, food bowl and water.

A nest box (plastic or wood) should be provided, loosely filled with straw or grass to replicate natural nesting burrows in soil cracks.^21^ Egg cartons can be used as “single‐use” housing (disposed of weekly/on cleaning day) (Figure 1)^18^ though care must always be taken to avoid contamination with any animal material that may pose a health threat to the colony (e.g., poultry fecal matter that may contain zoonotic parasites or bacteria that can infect marsupials e.g., *Toxoplasmosis* or *Salmonella*).^22^, ^23^, ^24^ Plastic is often easy to maintain hygienically in a laboratory setting as it can be sterilized in boiling water or washed using laboratory grade cage‐washers, though this is more labor‐intensive. Shredded office paper is used successfully as bedding, provided sources are of low‐toxicity.^25^ Shredded cardboard has been similarly used in laboratory settings, however, we observed this to cause (rare instances of) accidental castrations, so we recommend it be avoided for fat‐tailed dunnarts.

Captive dunnarts have been observed exhibiting stereotypic behaviors, particularly excessive grooming, or running in tight circles, when not provided with adequate enrichment. While the home range size of wild fat‐tailed dunnarts is unknown, they have been recorded to move at least 600 m,^14^ which is considerable for an animal weighing around 15 g. Running wheels can be provided to enclosure for enrichment (Figure 1). Captive fat‐tailed dunnarts were recorded voluntarily running >20 km in one night when provided with a running wheel. While this may be an indicator of an expansive wild home range, there are several contradictory explanations for voluntary wheel running in captivity^26^ and therefore, more research is required to allow full interpretation of this behavior. It is highly likely that this behavior is indicative of a captive environment not fulfilling the habitat complexities of wild environment, leading to a lack of adequate stimulation. When housing animals in a laboratory setting, sterility and enclosure replication are important, whilst balancing enrichment and promotion of wild behavior to avoid the establishment of stereotypic behaviors, which can lead to further physiological issues (e.g., influence on skull shape).^27^ Enrichment in a captive setting can be achieved through regular and novel provision of food items, opportunities to experience new environmental (habitat/cognition) and sensory (olfactory/auditory) stimuli, and social encounters. Hollow logs, bark shelters and hiding spaces should be provided for additional shelter, and native browse can also be provided as enrichment and replication of wild environment. Sand baths can be provided as enrichment and to assist dunnarts in performing natural shedding, digging and toileting behavior.^3^

#### 2.1.1 Light and temperature

If keeping fat‐tailed dunnarts at one temperature and one light cycle in captivity, without the aim of breeding, the temperature should aim to be at an average of 22 ± 3°C and a light cycle of 12 light: 12 dark hours, or 16 light: 8 dark is also appropriate. In the absence of natural lighting, light globes used in captivity should be full spectrum. Fat‐tailed dunnarts have trichromatic vision,^28^ meaning no light at night (or after daylight hours) is suitable, unlike many species where red light is appropriate. Light with different spectral wavelengths (both short‐ and long‐wavelength) have been shown to disrupt behavior and physiology in fat‐tailed dunnarts.^29^

To ensure wellness and maximize reproductive output, best practice is to replicate the natural conditions underlying this species' life history and breeding in the wild. Two distinct housing strategies are used, whereby two rooms under different artificial lighting and temperature regimes mirror summer and winter conditions:

1. Long light cycle (“summer”); (16 light hours: 8 dark hours) (16 L:8D) at 23 ± 3°C.
2. Short light cycle (“winter”); (eight light hours: 16 dark hours) (8 L:16D) at 20 ± 3°C.

See Reproductive Management section below for more information on maximizing the reproductive output of our colony, using lighting and temperature fluctuations to replicate seasonality.

Additionally, fat‐tailed dunnarts have adapted to harsh climatic fluctuations by developing the ability to enter torpor, which is a metabolic condition like hibernation, facilitating a reduction of energy loss.^30^, ^31^, ^32^, ^33^ This can be expected in captivity. Even when kept at a constant ambient temperature of 15°C fat‐tailed dunnarts have been recorded regularly entering torpor, and autonomously basking under heat lamps (15–31°C) when provided the option.^34^ Torpor generally occurs in wild fat‐tailed dunnarts when temperatures reach 6°C or lower,^30^ however reduced or unpredictable food supplies can also correlate to increased bouts of torpor.^32^

#### 2.1.2 Scent marking; captive consideration of wild biology

Scent marking is a crucial behavior to fat‐tailed dunnart life history, as with other Dasyurid marsupials.^35^, ^36^ This is conducted by using urine and feces to identify their territories and they often make latrine sites in both the wild and captivity. Latrines can be spot‐cleaned weekly to maintain hygiene. Full enclosure cleans can be conducted every 2–5 weeks by replacing all substrate flooring and providing new (clean) shelter sites.^17^, ^18^, ^24^, ^37^, ^38^ Old shelter sites should still be provided to allow the animals to move to clean shelters in their own time, minimizing stress and more closely aligning with wild behavior. In the wild, this species is predominantly solitary, though they may have overlapping territories.^14^ They regularly defecate through their nests and their territory as a method of communicating with other fat‐tailed dunnarts. In captivity, old shelter sites can be removed once fat‐tailed dunnarts have moved to the fresh, clean shelter. If their scent is removed too frequently, they will spend their time scent marking adding to stress levels.

#### 2.1.3 Determination of ideal enclosure substrates

Natural substrate and habitat complexity are always preferable for captive wildlife, as environmental elements closely reflecting wild habitat will reduce both morphological and behavioral adaptation to captivity.^39^

**FIGURE 2:**
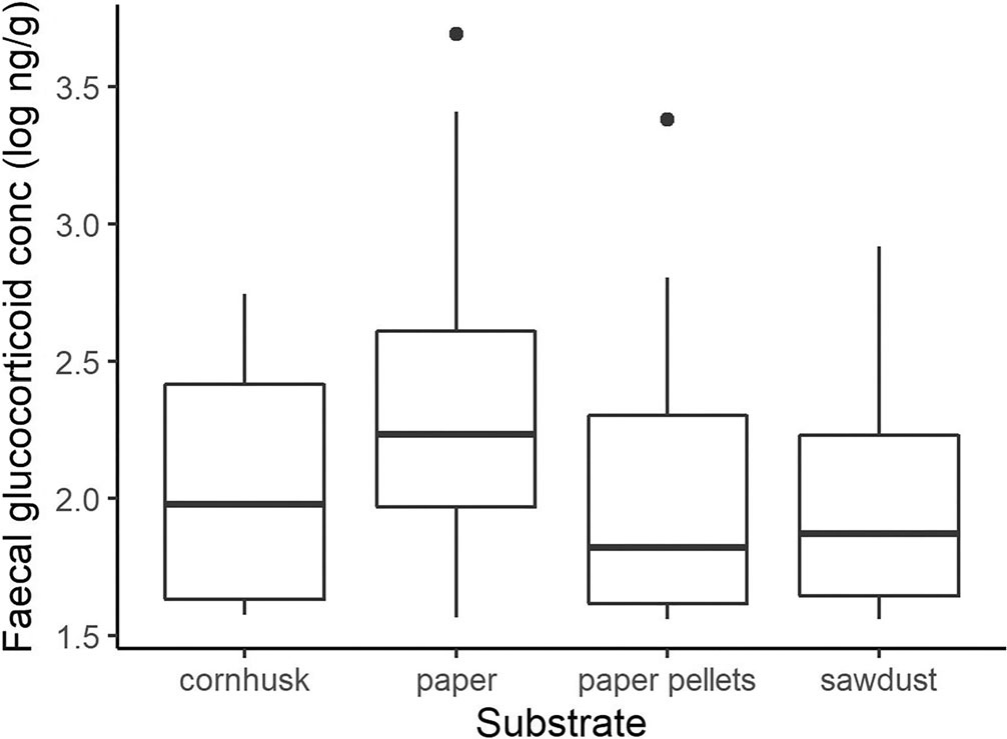
Effect of substrate transition on fecal glucocorticoid metabolite concentration across substrates (*p* < .05).

In a laboratory setting, commonly used substrates include sawdust, corn cob grit and compressed paper pellets (all of varying grades and coarseness) (Figure S1). Flat paper sheets can also be used (Figure 1), which allows easy hygiene maintenance, full view of animals (without feet or tail being obscured by substrate) for health checks, and easy observation of blood to identify birthing, or injuries. To investigate whether different substrates affect physiology, we compared baseline FGM concentrations across these four substrates (Figure 2). The type of substrate did not have a significant effect on FGM concentration (Figure 2; *χ* ^2^ = 5.24, df = 3, *p* = .15). Therefore, we consider other factors to provide a recommendation for the most appropriate captive management substrates. Sawdust, corn cob grit, and compressed paper pellets have the benefit of allowing “wild” behaviors like digging. However, all three loose substrates were ingested by fat‐tailed dunnarts (Figure S2), resulting in gastrointestinal impaction, and extreme cases, death. Additionally, cleanliness was more challenging to maintain on these substrates, which required more frequent, labour‐intensive cleaning required that was more disruptive to dunnarts (also see “Enclosure cleaning” below). In laboratory settings where enclosure substrates require frequent cleaning, an overall higher level of stress is induced for the fat‐tailed dunnarts. Hence, a situation‐specific balance must be achieved between wild habitat recapitulation and hygenic practicality, and we provide a discussion of the practical considerations of these substrates below.

#### 2.1.4 Sawdust substrates

MiniFlake sawdust can successfully be used as substrate^17^ (Figure S1a) for this species, and allows natural digging behaviors. However, sawdust has been linked to skin and respiratory disease in rodents,^40^, ^41^ with both significantly higher rates of sneezing and pathology scores found on rats kept on a similar substrate (aspen‐chip bedding^41^).

During birthing, blood spotting is not as easily observed on a sawdust substrate (as it is on paper), therefore is not recommended to use this substrate if precise days of pouch young birth is necessary (e.g., for embryology research^5^). Additionally, we observed (via necropsies of deceased animals, and analysis of fecal matter) sawdust to be consumed by animals and occasionally lead to gastrointestinal impaction (Figure S2a), so if used, it must be closely monitored.

#### 2.1.5 Corn cob grit substrates

Various sizes of corn cob grit are a commonly used substrate for laboratory rodent enclosures. However, mice (*Mus musculus*) have been observed ingesting this substrate,^42^, ^43^ and rats (*Rattus norvegicus*) housed on corn cob spent significantly less time in slow‐wave sleep, than aspen‐chip bedding (similar to MiniFlake sawdust).^44^ Fat‐tailed dunnarts trialed on corn cob grit (Figure S1b) occasionally showed redness under the feet, indicating irritation from the substrate. Analysis of fecal matter showed occasional consumption of this substrate, leading to gastrointestinal impaction (Figure S2b).

#### 2.1.6 Compressed paper pellet substrates

Compressed paper pellets (Alpha‐dri) (Figure S1c) provide greater absorption of waste compared to corn cob substrate, reducing the required frequency of cage cleaning for laboratory mouse enclosures.^45^ Intra‐cage ammonia levels were also significantly lower in ventilated paper pellet enclosures compared to corn cob,^45^ which is of particular significance to rodent husbandry, but less so to marsupials. Ammonia is a waste product, excreted as urea (nitrogenous waste) in all mammals.^46^ Given marsupials have lower nitrogen requirements than eutherians^31^, ^47^ and are generally held in less population‐dense enclosures, ammonia buildup is usually less of a problem for marsupials than rodents. While better at waste absorption, analysis of fecal matter showed that fat‐tailed dunnarts occasionally consumed the paper pellet substrate, leading to gastrointestinal impaction (Figure S2c).

#### 2.1.7 Flat paper sheets

The most effective substrate we have found in our laboratory are flat sheets of paper (Figure 1) or cardboard lining the enclosure floor.^18^ Increased cleanliness reduced the necessary frequency of cage‐cleaning, thus overall providing the lowest amount of stress to the fat‐tailed dunnarts. Provision of environmental enrichment with shredded paper, sand baths, or straw accommodates natural digging behaviors. In contrast to previously detailed substrates, no consumption and associated gastrointestinal impaction and mortality have been recorded with a sheet paper substrate. This substrate allows easy visualization of the entire animal (and all appendages) allowing ease of health checking. For example, the toes and tail are able to be seen in full (not buried in loose substrate) to allow checking for injuries, and/or potential signs of fighting between individuals. “Litter‐blood” can also easily be observed on paper when fat‐tailed dunnarts give birth, which is integral for management in a large captive colony, as it reduces the frequency of handling animals required for pouch checking, further reducing overall stress. The quantity of blood is very small, so it is much harder to observe on loose substrate. Sheets of paper or cardboard can be provided in layers, so that the top, heavily soiled layer is removed during cage cleaning, but some scent remains on the newly exposed layer. Thus, the importance of scent marking for this species is accounted for, reducing the stressful experience of a full enclosure clean when all olfactory stimuli are removed.

#### 2.1.8 Sand

Sand can be used as a substrate for this species, given its close alignment to wild habitat and encouragement of wild behaviors (e.g., digging burrows for nesting, and foraging for invertebrate prey items). However, sand has been linked to gastrointestinal impaction^48^, ^49^ and irregular tooth wear^50^ in a variety of captive mammals after multiple generations bred in captivity, so this needs to be monitored closely. Sand is also challenging to manage practically and hygienically in a laboratory setting, therefore is not recommended in this captive management context.

#### 2.1.9 Fecal cortisol levels are significantly impacted by substrate transition

**FIGURE 3:**
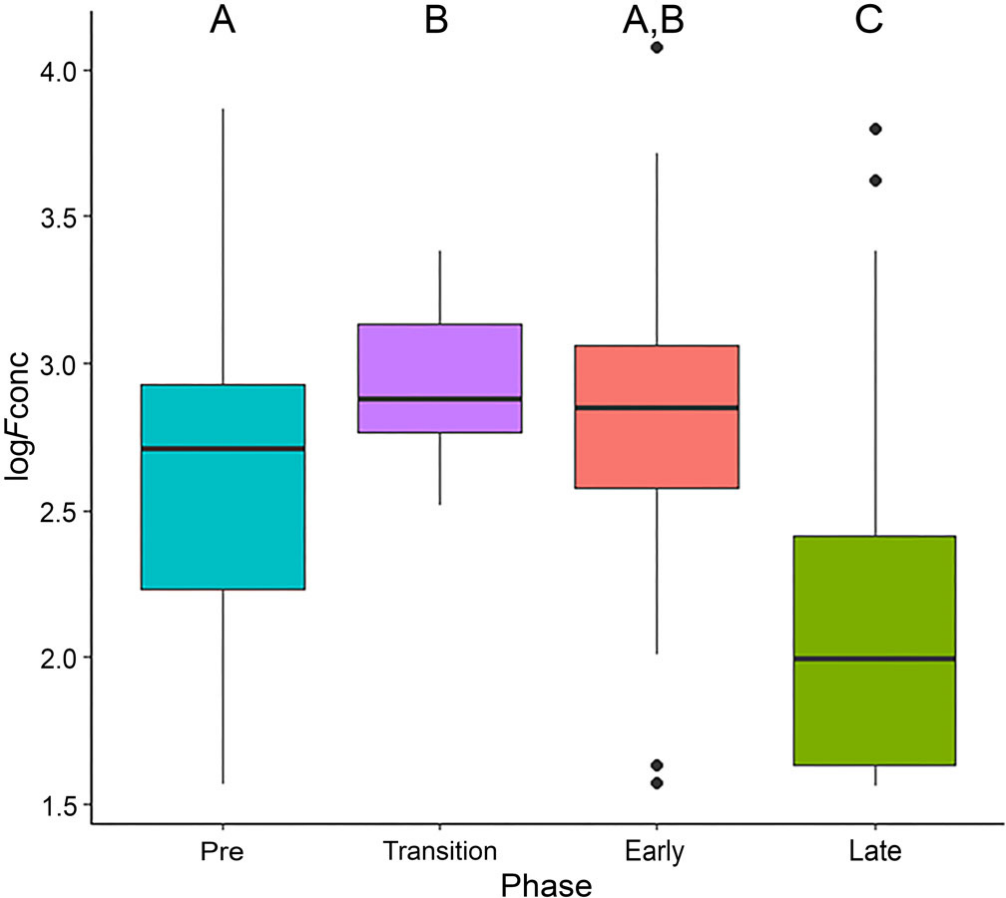
Effect of substrate transition on fecal glucocorticoid metabolite concentration. Transition onto new substrate was broken into four phases: Pre = before transition (4 days); Transition = 2 days of mixed substrate; Early = first 10 days of entirely new substrate; and Late = remainder of time (23 days). Data are pooled from all substrates and sexes pooled. Groups that do not share a common letter are significantly different (*p* < .05).

**FIGURE 4:**
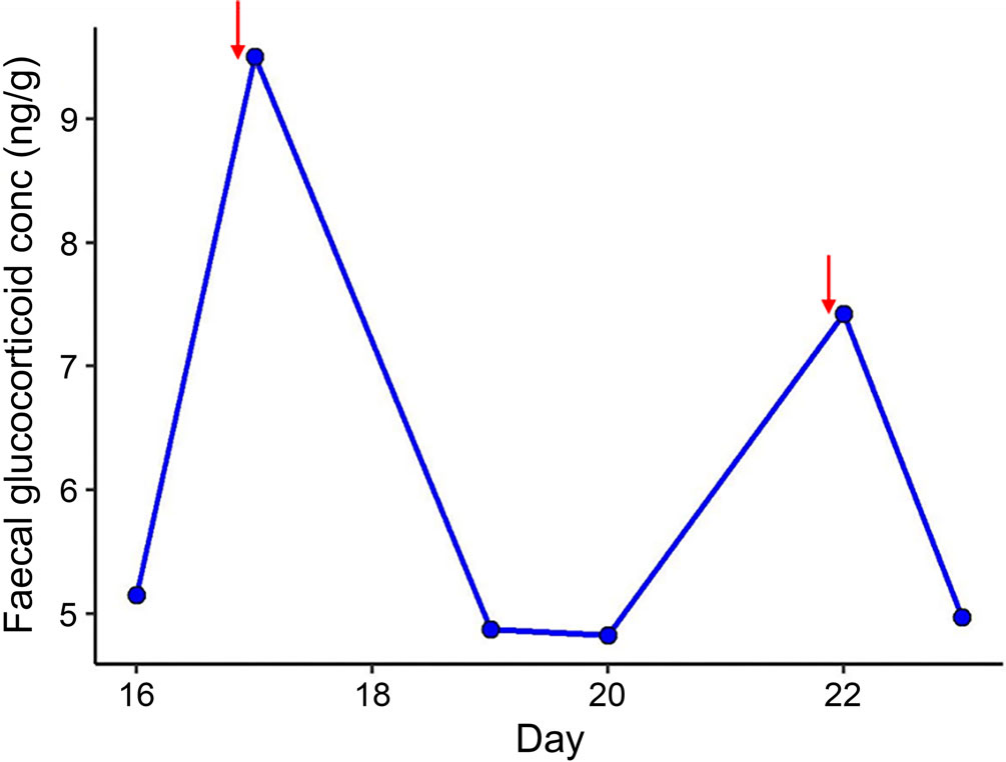
Effect of cage cleaning on fecal glucocorticoid metabolite concentrations (single enclosure 276,440). Red arrows indicate days when the cage was cleaned in the morning and the sample was collected in the afternoon.

To investigate how major changes to the enclosure impact dunnart physiology, we monitored FGM concentrations during a transition from one substrate to another. We transitioned enclosure substrates from plain paper substrate to either sawdust, corn cob grit or paper pellets. FGMs were measured daily for 4 days prior to substrate transition, and up to 3 weeks following substrate transition. Substrate transition had a significant effect on FGM concentrations (*χ* ^2^ = 121.81, df = 3, *p* < .001), with levels significantly higher during and after the transition (Figure 3) compared to pre‐transition. After 10 days, FGM concentrations returned to baseline and were significantly lower than the transition and early‐acclimation phase (Figure 3). There was no significant difference between males and females (*χ* ^2^ = 1.81, df = 1, *p* = .18). Fat‐tailed dunnarts demonstrated a similar physiological response to substrate transition for all substrates trialed, and no significant interaction between phase and substrate was detected. (Figure 4; *χ* ^2^ = 0.012, df = 2, *p* = .99). There was also no significant effect of substrate type on baseline FGMs (early and late phase; Figure 3). Therefore, we recommend avoiding major changes to the enclosure unless necessary. Slow introduction of new substrates is recommended to allow acclimation to the substrate and learn to avoid consuming, if it becomes stuck on food.

### 2.2 Husbandry

#### 2.2.1 Diet in captivity

Fat‐tailed dunnarts are nocturnal and predominantly insectivorous, however in the wild have been observed to kill small vertebrates including house mice.^51^ Small lizards have also been found in fecal samples.^52^ Key dietary items in wild environments include both hard‐ and soft‐bodied invertebrates, including spiders, beetles (adult and larval form), slaters, centipedes, termites, ants, earthworms, grasshoppers, crickets and earwigs.^52^, ^53^ The species has adapted to store fat in their tail as an energy reserve for less‐desirable environmental conditions, when invertebrate food sources become scarce.^30^, ^53^

Adult fat‐tailed dunnart require 10 g of food a day, and up to 40 g per day for breeding females.^16^, ^19^ A red meat‐based diet supplemented with live vertebrates best mimics their natural diet.^54^ Any raw meat being fed is recommended to be frozen for 4 weeks prior to feeding, to minimize the risk of bacterial or parasite contagion (e.g., Toxoplasmosis) which can cause considerable mortality.^24^ The species can be successfully reared in captivity on a wet cat food mix. In many captive colonies, *Whiskas* (*Mars Australia Pty Ltd, Australia*) dry cat food (beef and lamb) has been soaked in hot water, then mixed with cans of *Whiskas* wet cat food (Jellymeat loaf), combined with *Wombaroo* Small Carnivore Mix (*Wombaroo Food Products, Australia*) (three parts powder [15 g] to two parts water [10 mL]).^7^, ^16^, ^19^, ^37^, ^38^, ^54^, ^55^ Over time, we found that the meat content of *Whiskas* products differed, leading to fluctuating levels of protein consumed by the fat‐tailed dunnarts. *Advance Kitten* (*Advance Pet Food, Australia*) dry biscuits and *Hills Adult Cat Science Diet* (*Hill's Pet Nutrition, Inc, USA*) have successfully replaced *Whiskas* in The University Of Melbourne fat‐tailed dunnart colony diet since 2022, to increase nutritional content and ensure adequate levels of protein.^18^ Royal Canin Recovery Feline/Canine food (*Royal Canin SAS, France*) can serve a suitable replacement. The addition of *Vetsense* Paraffin Oil (*Vetsense, Australia*) has been successfully used to reduce formation of furballs and gut impaction since 2021 (*unpublished data*), and *Wombaroo—The Good Oil* has also been added to increase essential vitamins and fatty acids.^18^ *Wombaroo Small Carnivore Mix* can represent up to 80% of the dunnarts' total diet.^56^ An alternative diet with similar nutritional content (readily used in captive colonies) includes a mix of meats, egg and invertebrates or *Wombaroo Small Carnivore Mix*.^56^ Calcium carbonate can be added to diet to ensure bone health.^55^ Hard dietary items are recommended to avoid divergence in skull morphology.^27^ For example, increased supplementary feeding of invertebrates with hard exoskeletons (ie. Woodroaches, beetles, spiders and centipedes) may help to overcome this issue. Mealworms are a favourite invertebrate food items of fat‐tailed dunnarts, however they are high in fat and therefore should be provided in moderation.

Water should be available ad libitum. Cage bird seed dispensers are most appropriate for water presentation and are preferable to rodent sipper tubes which require training and deviation from wild behavior. Pentavite multivitamin (*Pentavite Pty Ltd, Australia*) can be added to water (e.g., two drops per 125 mL bird seed feeder) to boost nutrition,^16^, ^51^ however if adequate nutrition is met through diet, this is not necessary.

#### 2.2.2 Enclosure cleaning is a significant stressor

It is generally accepted that enclosure cleaning is stressful for dasyurid species, particularly because of their reliance on scent marking as a form of inter and intra‐specific communication^35^, ^36^ (and cleaning involves removal of scent). However, there is a lack of empirical evidence regarding the impact of cage cleaning on physiology. To better understand how cage cleaning impacts fat‐tailed dunnarts, we monitored changes in FGM concentrations in response to cleaning events. Fecal samples were collected daily for 2 weeks. Cages were cleaned thoroughly once per week, and an effort was made to minimize any other stressors. Cleaning occurred first thing in the morning and samples were collected in the afternoon. Accounting for excretion lag time, measured FGM concentrations would reflect events that occurred that morning.

FGM concentrations were significantly higher on days when the cages were cleaned compared to non‐change days (*χ* ^2^ = 28.35, df = 1, *p* < .001). Cleaning caused an acute, transient increase in FGMs, followed by a return to baseline within 24 h (Figure 4). No sex‐ specific response to cage cleaning was observed (Figure S3).

Repeated exposure to an acute stressor can be just as detrimental as chronic exposure to stress.^58^ Therefore excessive cleaning should be avoided, with cleaning no more than once a week.

#### 2.2.3 Refined handling procedures to minimize animal stress

**FIGURE 5:**
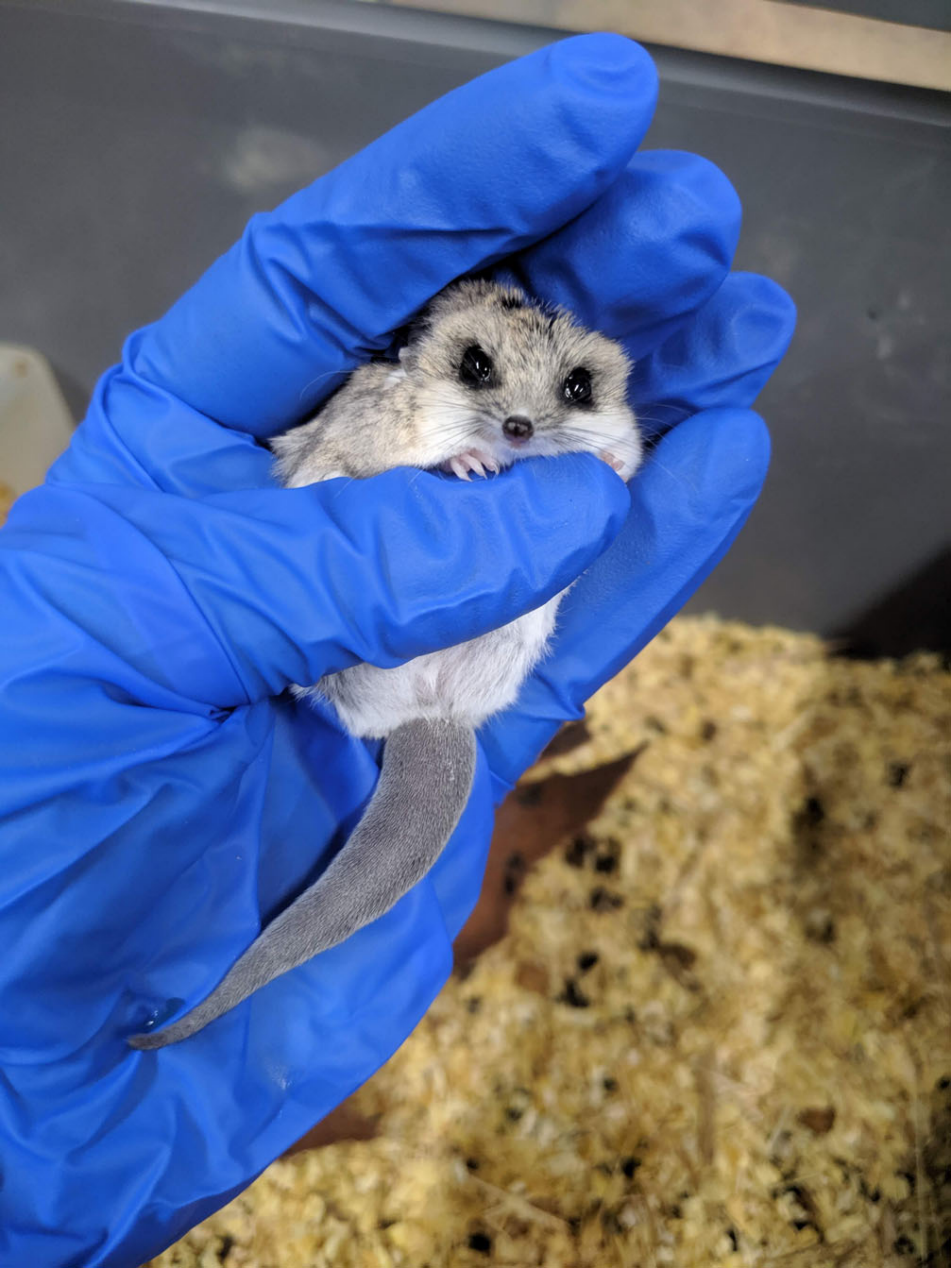
Correct handling of fat‐tailed dunnart, showing fingers cupped around body, with thumb under chin, and tail unrestrained.

Dunnarts can be successfully captured by cupping a hand over animal, then moving the thumb under the animal's chest to hold firmly, but not tightly. Allow fingers to sit over the animal's neck and head, with thumb resting under the dunnart's chin (Figure 5). If the animal feels supported but not threatened with injury, it is more likely to remain calm and move less. Always use the least amount of restraint necessary and hold animal in a cotton catch‐bag if practical. Do not grab or hold fat‐tailed dunnarts by the tail for risk of injury, including sloughing of skin.^54^ If bitten by a dunnart, to avoid injuring the jaw, do not jerk hand away and instead encourage dunnart to release independently. Minimizing loud noises or sudden movements around the animals is recommended whenever possible to reduce stress, particularly when handling. “Scruffing”^54^ should be avoided unless necessary for an experimental procedure, as this has been found to cause unnecessary stress, or injury (in mice, though the result is considered transferable to other small mammals).^59^ Accordingly, despite being routinely utilized in laboratory mice, this method is no longer referenced as an appropriate handling method.^20^

Like most Dasyurids, fat‐tailed dunnarts are sensitive to olfactory stimuli (e.g., reliance on scent marking for communication), which needs to be considered when handling dunnarts. Care should be taken to avoid antagonism between animals, through the distribution of scent. Therefore, gloves should be worn and changed between individuals (particularly males) to avoid the scent of other individuals inducing stress or aggressive behavior. Alternatively, between handling of distinct individuals, gloves can be cleaned with ≥70% ethanol or F10FC Veterinary Disinfectant and allowed to minimize scent carry over.

#### 2.2.4 Health checks

Each individual animal should be sighted at least twice a week (usually during enclosure cleans). We developed body condition assessment matrices to assess health in both juvenile (Table 3) and adult (Table 4) fat‐tailed dunnarts. Key features to assess for signs of ill health include weight, posture, tail thickness, coat and eye scores. A healthy fat‐tailed dunnart is sitting at a stable, or gaining weight, has active, alert behavior and upright posture, a swollen and bulbous tail, is well groomed with a thick, full coat (no hair loss), and wide, glassy and alert eyes. An unwell dunnart may present rapid weight loss, a hunched appearance, hair standing on end, cool to touch, severely thin tail, greasy or ruffled fur, hair loss, blood or scabbing present, and eyes might be squinting, closed or presence of discharge. Many signs of ill health can easily be confused with torpor (hunched posture, body trembling, hair standing on end, wobbly movement etc), therefore it is only after these symptoms persist after handling for a minute (in which time dunnart should become warmer and alert) that this becomes a true indicator of their body score.

**TABLE 3:** Independent juvenile (growing) fat‐tailed dunnart (70–200 days old) body condition assessment matrix.

Health determinants	Criteria and scoring			
Weight score	Rapid weight loss/hips protruding	Weight loss	Stable weight	Gaining weight
	1	2	3	4
Posture score*	Hunched/hair on end/frozen movement/cool to touch/limp	Slow, unbalanced movements, cool to touch	Slightly abnormal behavior/less alert	Normal, active, alert behavior
	1	2	3	4
Tail score	Injury/severely thin	Thin, limp	Thin	Swollen/bulbous
	1	2	3	4
Coat score	Greasy/ruffled fur/substantial hair loss/wet/blood/scabbing present	General hair loss/greasy or ruffled fur/wet/some blood/scabbing present	Slight hair loss (at tail base)/not as well groomed	Well groomed, full thick coat
	1	2	3	4
Eye score*	Eyes closed/significant discharge	Squinting/dull/discharge present	Alert/slight squint/slight discharge	Wide, black, glassy, and alert
	1	2	3	4
Accumulative total (out of 20)				
Total	0–5	6–10	11–15	16–20
Body score	1 (Poor)	2 (Low)	3 (Moderate)	4 (Good)

**TABLE 4:** Adult (fully grown) fat‐tailed dunnart (>200 days old) body condition assessment matrix.

Health determinants	Criteria and scoring			
Weight score	Rapid weight loss/hips protruding/visible skull definition	<12 g/losing weight/visible skull definition	12–14 g/stable	15–20 g/stable
	1	2	3	4
Posture score*	Hunched/hair on end/frozen movement/cool to touch/limp	Slow, unbalanced movements, cool to touch	Slightly abnormal behavior/less alert	Normal, active, alert behavior
	1	2	3	4
Tail score	Thin, limp/injury	Thin–moderate	Moderate	Fat, healthy
	1	2	3	4
Coat score	Greasy/ruffled fur/substantial hair loss/wet/blood/scabbing present	General hair loss/greasy or ruffled fur/wet/some blood/scabbing present	Slight hair loss (at tail base)/not as well groomed	Well groomed, full thick coat
	1	2	3	4
Eye score*	Eyes closed/significant discharge	Squinting/dull/discharge present	Alert/slight squint/slight discharge	Wide, black, glassy, and alert
Accumulative total (out of 20)				
Total	0–5	6–10	11–15	16–20
Body score	1 (Poor)	2 (Low)	3 (Moderate)	4 (Good)

### 2.3 Reproductive management

#### 2.3.1 Replication of seasonality using light cycling and temperature to improve captive breeding

Breeding season (in wild populations) is between July and February, where female oestrous aligns with the end of winter.^14^ First litters of the season appear in July to October, aligning with spring when their invertebrate food source is abundant in the environment.^60^ Females will enter a second oestrous after weaning the first litter, and can have a second litter between November and February. That said, this occurs infrequently as it is dependent on the availability of males, therefore while up to three litters is possible, this is very rare.^61^ Litters usually average 7 pouch young.^5^ An average of 10% of recorded litters are lost before the age of weaning,^16^ but females can ovulate up to 14 days after losing young,^61^ and can successfully rear a litter from their second oestrous cycle despite losing the first litter.^2^

When a female has weaned her young she will undergo a post‐weaning estrous in 1–2 days and can mate in 5–9 days. Females can be paired with males immediately after weaning her young, if further litters are required.^16^ If a second estrous period is missed when attempting to breed in captivity, females can be returned to short light cycle for 2 weeks to prompt re‐entry into oestrus as described above. Females are likely to exhibit a reduction in litter size over time, with younger females producing larger litters.^62^

To maximize the reproductive output of our colony, lighting and temperature fluctuations need to be used to replicate seasonality. Females can be stimulated into ovulating at the cessation of perceived Winter (time spent in short light cycle room), which aligns with natural reproductive timing in the wild. Fat‐tailed dunnarts have adapted to enter oestrous at the end of winter so they give birth to young in Spring. In the wild, young are then weaned in spring/summer during times of high invertebrate abundance, increasing the survival rate.^60^

In attempt to mimic wild life‐history, male and female pairs can be kept in short light cycle (8 L:16D; 20 ± 3°C), and then can be moved to long light cycle (16 L:8D; 23 ± 3°C). In addition to the shift to increased temperature and perceived day length, the addition of extra invertebrate prey items provided at this time can help simulate the end of Winter and start of Spring, representing the start of the fat‐tailed dunnart's wild breeding season. Females enter their reproductive state of oestrous ~20–30 days after they experience an increase in day length and temperature (after the change from “Winter” to “Summer” room). Best breeding output has been achieved by pairing both day length and temperature change, not just one (e.g., day length).

Once pouch young are weaned, females should move from natural lights or artificial long light cycle (16 L:8D) and 23 ± 3°C to short light cycle (8 L:16D) and 20 ± 3°C for ~3 weeks. In the third/final week of short light cycle time, females can be paired with a male, as long as they are of similar size and of similar reproductive maturity. This ensures animals are aligning with wild biology and experience seasonal cues in lighting and temperature to promote breeding, in the absence of natural/wild environmental cues.

Further details of critical life history and morphological trait development are shown in Tables 1 and 2. See Newton et al.^5^ for further information on reproductive development.

#### 2.3.2 Monitoring, husbandry, and detection of pregnancy in dunnarts

Using this strategy, we have additionally established monitoring methods for husbandry, detection of pregnancy and embryo collection in the fat‐tailed dunnart.^5^ In alignment with wild life‐history, females should be paired with males for breeding as soon as reproductively mature, to capture the female's first oestrous period. Oestrous and ovulation are identifiable through cyclical weight fluctuations^5^ and supplemented by monitoring the cornified epithelial cells present in the urine of the female.^5^, ^63^, ^64^ Spermatozoa may also be seen after mating, but is not as reliable in predicting pregnancy as daily weight monitoring. To reduce the cumulative stress experienced by the dunnart, the repetitious handling required for both weight and urine cytology should only be performed when necessary for experimental or management outcomes.

#### 2.3.3 Monitoring for pouch young

Birth typically occurs after 14 days,^7^, ^65^ so 15 days after presumptive mating, the pouch can be checked for the presence of young.^16^ Behavioral changes often accompany the presence of pouch young. Females can exhibit increased defensive behavior when they are pregnant or are carrying pouch young,^3^ so males can often behave more erratically or avoid female. Additionally, if animals are housed on a white paper substrate, observation of birthing blood is indicative of the presence of pouch young. As soon as pouch young are identified the male should be removed, and females with pouch young should be housed individually in their home enclosure, ensuring the continuity of olfactory stimuli. Food also should be increased from 10 to 40 g/day to sustain the mother and support lactation. Minimize disturbance as much as possible. Until their young are weaned, mothers' enclosures should be cleaned at a maximum of once a week, as this is a major stressor and can impact reproductive success.

**FIGURE 6:**
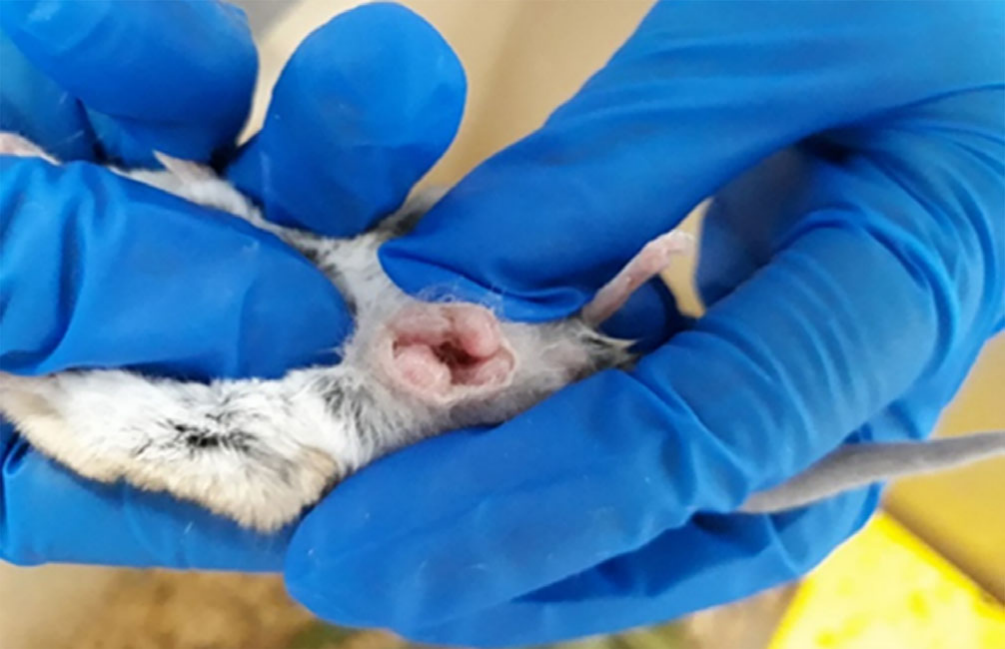
Correct handling of female fat‐tailed dunnart for pouch check, showing young pouch young protruding from pouch.

The pouch of a nulliparous (not reproductively active) female will appear tight, dry and full of hair. The pouches of parous females (pregnant or in oestrous) appear looser (easier to open), hairless, and pink as the female cleans it in preparation for young.^1^ Pouch checks can be conducted by using the cupping capture technique (described above), then gently rolling the animal onto her back to expose the stomach. To secure the animal in one hand, it is easiest to hold animal with her tail towards the top of your fingers, placing your thumb on the dunnart's chest, and keeping the head towards the palm of your hand. This position should allow the other hand to gently open the pouch, located low on the female's abdomen (Figure 6). Pouch young will be attached to nipples and therefore are usually obvious inside the pouch, however this depends on developmental stage. Minimize time spent pouch‐checking to minimize handling stress. Increased stress may have repercussions for the survival of pouch young,^66^ particularly as some marsupials have been known to eject pouch young in response to a perceived threat, allowing the mothers a better chance to survive if avoiding a predator.^67^ For this reason, do not attempt to count or sex pouch young at this time unless absolutely integral. Not only does this reduce stress for mother, but number of pouch young often changes over time. Some pouch young may be rejected by the mother due to slow development.

#### 2.3.4 Growth and development of pouch young

Dunnart prenatal development occurs over a 14‐day gestation, with organogenesis occurring rapidly from day 10.^5^ At birth, the highly altricial fat‐tailed dunnart neonate lacks an ossified skeleton, and is instead entirely cartilaginous, with their first bones ossifying within the first 24 h.^7^ Their mouths are entirely fused except for a small opening allowing attachment to mother's nipple.^7^ The young dunnart's jaw hinges grow around the nipple until around 30 days old when teeth erupt.^7^ This means they cannot easily detach from the mother. Given the internal development of these young occurs external to the mother's body, they can be easily injured or damaged and extra care must be taken if handling a mother is necessary.

Pouch young will be protruding from the mother's pouch by 37 days, allowing visualization without pouch checking. Once the young detach from the nipples, the mother will leave them in the nest at 59–63 days (Table 2). At this stage an alternative nest box can be provided to allow the mother and young extra space. Pouch young need to be weaned from their mother at 65–70 days old. By this stage young will be leaving the nest, exploring individually and eating solid food (Table 2). If weaning does not occur, the mother will likely become territorial towards young and may exhibit defensive/aggressive behavior. This behavior aligns with the life history of this species in the wild, given they are predominantly solitary,^14^ do not live in family groups and separate after weaning.

While up to 70% of dunnarts will nest‐share when necessary (for warmth during the non‐breeding season),^1^ care needs to be taken to minimize provocation of increased aggression, when co‐housed in captivity. When cohoused, animals should be monitored closely for behavioral changes. Following weaning, same‐sex siblings can remain housed together until they are paired with mates. At this stage in the wild littermates will have all separated from siblings and moved away to establish individual territories,^62^ leading to an increase in territorial behavior when presented with mates. Thus, after mating encounters, males should be housed individually to prevent aggression which can lead to deaths. Female siblings can continue being housed together or with a male (multiple females to one male, or 1:1 male and female, or multiple females and no male) provided they have adequate space and shelters to avoid each other. Aggression can occur when one female enters estrous and behavioral changes ensue.^3^ Similarly, if there are too many individuals in an enclosure and a stressor occurs, heightened aggression can be expected. Animals that were previously living contentedly together (e.g., same‐sex groups) may experience rapid behavioral change, which can lead to death if not managed appropriately.

In the wild, dunnarts exhibit mate choice^68^ therefore females should be given the opportunity to “choose” a mate. This can be achieved by offering a female the soiled bedding of two genetically suitable males, and assessing the interaction time with individual bedding as an indicator of preference. A lack of breeding success may not necessarily indicate infertility, so if a mating pair have not produced pouch young in 6–8 weeks, re‐pair the individuals with alternative mates to account for potential mate incompatibility. However, if a male is unsuccessful in producing young after being paired with multiple females, his urine should be checked for the presence of sperm, indicating level of fertility.^2^

Population management is necessary to manage captive breeding. A diary should be established to track the reproductive cycles of each female. Based on date of pouch young being found in a pouch, their date of birth can then be used to calculate the date they need to be weaned from their mother (70 days), when they need to move from long to short light cycle to stimulate breeding (100 days), date of approximate reproductive maturity (females: 85–120 days, males: 210 days), etc. Strict records regarding an animal's parents and mates need to be kept, to track and ensure genetic outbreeding, with avoidance of breeding related individuals. Genetic analyses can be conducted to construct pedigree charts for the colony, tracking the presence or absence of genetic relatedness, as well as the presence of particular genes. At the very least, a rotational breeding system can be implemented whereby each animal is designated a color, and only specific color males and females can be mated, resulting in progeny of another color.^69^ Captive populations are often established with a small number of individuals from a population. If those individuals show detrimental genetic traits in common, animals with the least relatedness and least likelihood of passing on detrimental genes should be bred. Similarly, selecting individuals with rare alleles for breeding, retains genetic diversity across captive‐bred generations.^70^ For large scale endangered species captive breeding, genetic matrices can be developed to track kinship (genetic relatedness) and establish better suited partners through being more unrelated.

## 3 SUMMARY

Here we present a consolidated resource and discussion of best practices for the utilization of fat‐tailed dunnarts as a model species in marsupial research, emphasizing the imperative assessment of potential impacts of captivity on their morphology and behavior. We highlight the necessity of incorporating the species' natural biology into captive husbandry practices, encompassing considerations for enclosure requirements, substrate selection, and provision of natural habitat elements. We detail guidelines for feeding practices, encapsulating the importance of a diet that mirrors their natural preferences, and emphasize the significance of minimizing stress during handling, particularly in breeding situations. Scent marking behavior is a crucial aspect of fat‐tailed dunnart life history, and excessive cleaning in captivity can adversely affect reproductive success. Furthermore, lighting simulating seasonal cycles is important to induce and maintain reproductive behaviors, with further insights into monitoring reproductive cycles, conducting pouch checks, and implementing effective weaning practices. Finally, by incorporating strategies for population management, including genetic diversity preservation and rotational breeding, our best practices present a holistic perspective on the captive management and breeding of fat‐tailed dunnarts, emphasizing the alignment of practices with their natural behaviors for sustained success in laboratory settings.

## 4 EXPERIMENTAL PROCEDURES

### 4.1 Study population

Captive fat‐tailed dunnarts were held at The University of Melbourne. Animals were housed in temperature and light‐controlled rooms (designed to replicate natural seasonality), in 36 L plastic tubs (45 × 30 × 25 cm; length × width × height) with custom‐made stainless steel ventilated lids. All individuals in this study were cohoused with same‐sex individuals. Males (reproductively immature) were cohoused with littermates only. Some females were housed with littermates, however, some reproductively mature females were also housed with other female conspecifics.

### 4.2 Substrate comparisons

A total of 12 “stock” enclosures were sampled. Within the 12 “stock” enclosures, four enclosures (two female enclosures, two male enclosures) transitioned from paper sheets to each of the experimental substrates; corn cob grit, sawdust or paper pellets (Table 1; Figure S1; Table S1). For the first 2 days of the transition, animals were given a combination of paper sheets and new substrate, before being shifted completely to the new substrate. In total, samples were collected from eight females and six males exposed to corn cob grit, nine females and six males exposed to sawdust and eight females and eight males exposed to paper pellets. During this time, enclosures were cleaned thoroughly once per week first thing in the morning (~7:00 am−9:00 am).

Small granule (3 mm) corn cob grit was sourced from Corncobology and is a low dust, absorbent substrate considered optimal for small animals. MiniFlake sawdust (5–10 mm) was sourced from Pollard's Sawdust Supplies, and is a highly absorbent substrate processed from kiln dried, pine wood‐shavings. Paper pellets were obtained from Alpha‐dri, and are 5 mm^2^ pieces of sterile, virgin paper pulp cellulose.

### 4.3 Fecal sample collection

Fecal samples were collected daily from 4 days before to 3 weeks after the substrate transition. Fresh fecal samples were collected from each enclosure between 2:00 pm and 5:00 pm. A mix of fresh fecal deposits were collected from around each enclosure in attempt to get a representative sample of the different individuals in each enclosure. Fecal samples were collected using ethanol‐sterilized forceps, and stored at −80°C prior to analysis. Based on the steroid excretion lag time for dunnarts, FGM concentration would reflect events that occurred ~5 h earlier. Hence, the timing of sample collection aligned with 5–6 h post stressor, and was conducted in the same order the cages were cleaned, to ensure timing alignment. Samples collected during the transition onto the new substrate were categorized into one of four phases.

### 4.4 Steroid extraction and enzyme‐immunoassay

To extract steroid metabolites, fecal samples were weighed to 0.02 g (0.019–0.021 g), and 1 mL of 80% ethanol was added to each sample. Samples were vortexed and shaken on an orbital shaker overnight. The following day, samples were centrifuged for 5 min at 5000 rpm, and the supernatant was decanted into separate vials.

FGM concentrations were quantified using a cortisol, enzyme‐immunoassay (Arbor Assays, USA, product # ISWE002).

The assay was run according to the manufacturer's protocol, as previously described.^71^ Briefly, 96‐well microtiter plates were coated with goat anti‐rabbit IgG (Arbor Assays, USA, A009) and incubated at 4°C overnight. To run the assay, plates were washed and loaded with 50 μL of standard, control, buffer or diluted sample to appropriate wells, followed by 50 μL cortisol horseradish peroxidase solution and 50 μL cortisol antibody solution. Plates were shaken for 2 h at room temperature, then washed 4 times with 300 μL wash solution. One‐hundred microliter TMB solution was added to each well, and after 30 minutes of incubation at room temperature plates were stopped using 50 μL of stop reagent. The absorbance was read at 450 nm (650 nm reference filter) using a SPECTROstar Nano plate reader (BMG LABTECH). All samples were run in duplicate. The assay was biochemically validated by demonstrating parallelism between the standard curve and pool of diluted sample extracts.

### 4.5 Statistical analysis

Data analysis was conducted in R v4.1.1. FGM concentrations were log transformed to meet model assumptions of a normal distribution and homoscedasticity. Statistical significance was set at *α* = .05.

To investigate the effect of substrate transitions on FGM concentrations, data were grouped into four phases: “Pre” = before transition (4 days); “Transition” = 2 days of mixed substrate; “Early” = first 10 days of entirely new substrate; “Late” = remainder of time on new substrate (23 days). Using a linear mixed model (package lme4) FGM concentration was modeled as a function of the phase of the transition and sex. Enclosure ID was included as a random effect to account for repeated sampling.

To test whether substrate type affected physiology, we compared baseline FGM concentrations across the four substrates. To avoid confounding influences on FGM measurements, we excluded the “Transition” and “Early” phases since FGM concentrations were significantly elevated, as well as days that cages were changed (see below). FGM concentration was modeled as a function of substrate type and sex, with enclosure ID as a random effect.

Finally, we examined the effect of cage cleaning on FGM concentrations. The dataset for this analysis was limited to >10 days after the substrate transition to avoid confounding effects of this event. It includes two–three cage cleans per cage for 12 enclosures. We used a linear model to test whether there was a difference between days when the cages were cleaned compared to days when cages were not cleaned. We also included sex in the model and a random effect for enclosure ID.

## CONFLICT OF INTEREST STATEMENT

The authors do not declare any conflicts of interest.

## Supporting information


**Data S1.** Supporting information.
