# Supplementary material for: Breeding fat‐tailed dunnarts (Sminthopsis crassicaudata) in captivity: Revised practices to minimize stress whilst maintaining considerations of wild biology
Source: Dev Dyn. 2025 Feb 2;254(2):189–204. doi: 10.1002/dvdy.755 (PMC11809136; doi:10.1002/dvdy.755)
Supplement: Supplementary file 1 — Data S1. Supporting information. [file DVDY-254-189-s001.docx]

**Breeding fat-tailed dunnarts *(Sminthopsis crassicaudata)* in captivity: revised practices to minimize stress whilst maintaining considerations of wild biology**

**Supplementary information**

| Enclosure | No. dunnarts | Sex | Experimental substrate |
| --- | --- | --- | --- |
| 1 | 4 | Male | cornhusk |
| 2 | 4 | Male | sawdust |
| 3 | 2 | Male | cornhusk |
| 4 | 5 | Male | paper pellets |
| 5 | 2 | Male | sawdust |
| 6 | 5 | Male | paper pellets |
| 7 | 5 | Female | cornhusk |
| 8 | 6 | Female | sawdust |
| 9 | 3 | Female | sawdust |
| 10 | 4 | Female | paper pellets |
| 11 | 3 | Female | cornhusk |
| 12 | 4 | Female | paper pellets |
| 12 | 4 | Female | paper pellets |

Table 1: Number and sexes of dunnart individuals held in each enclosure, exposed to various experimental substrates.


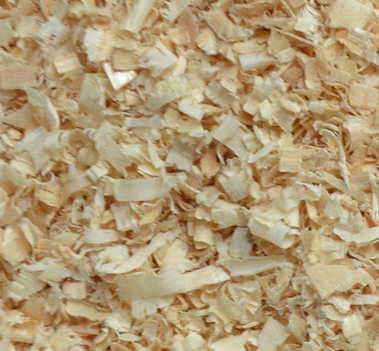

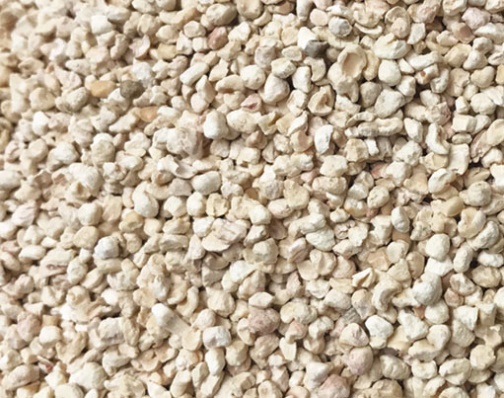

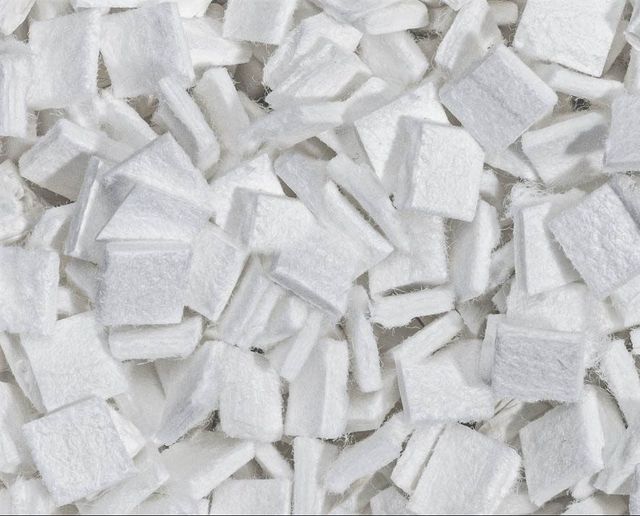


a)

b)

c)

**Figure 1:** Experimental floor substrates used to line fat-tailed dunnart enclosures, including a) MiniFlake sawdust b) corn cob grit and c) Alpha-dri paper pellets

**
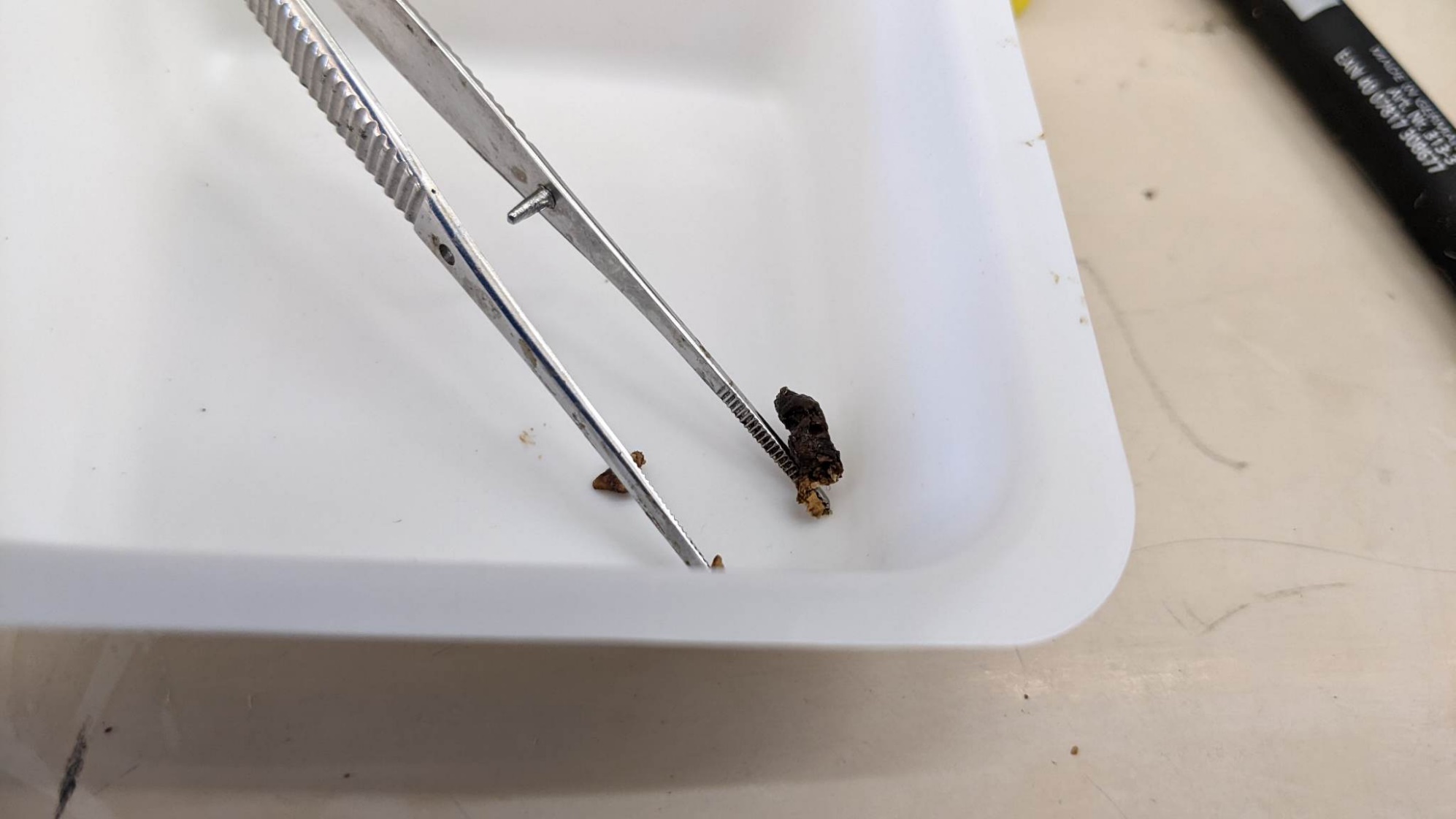

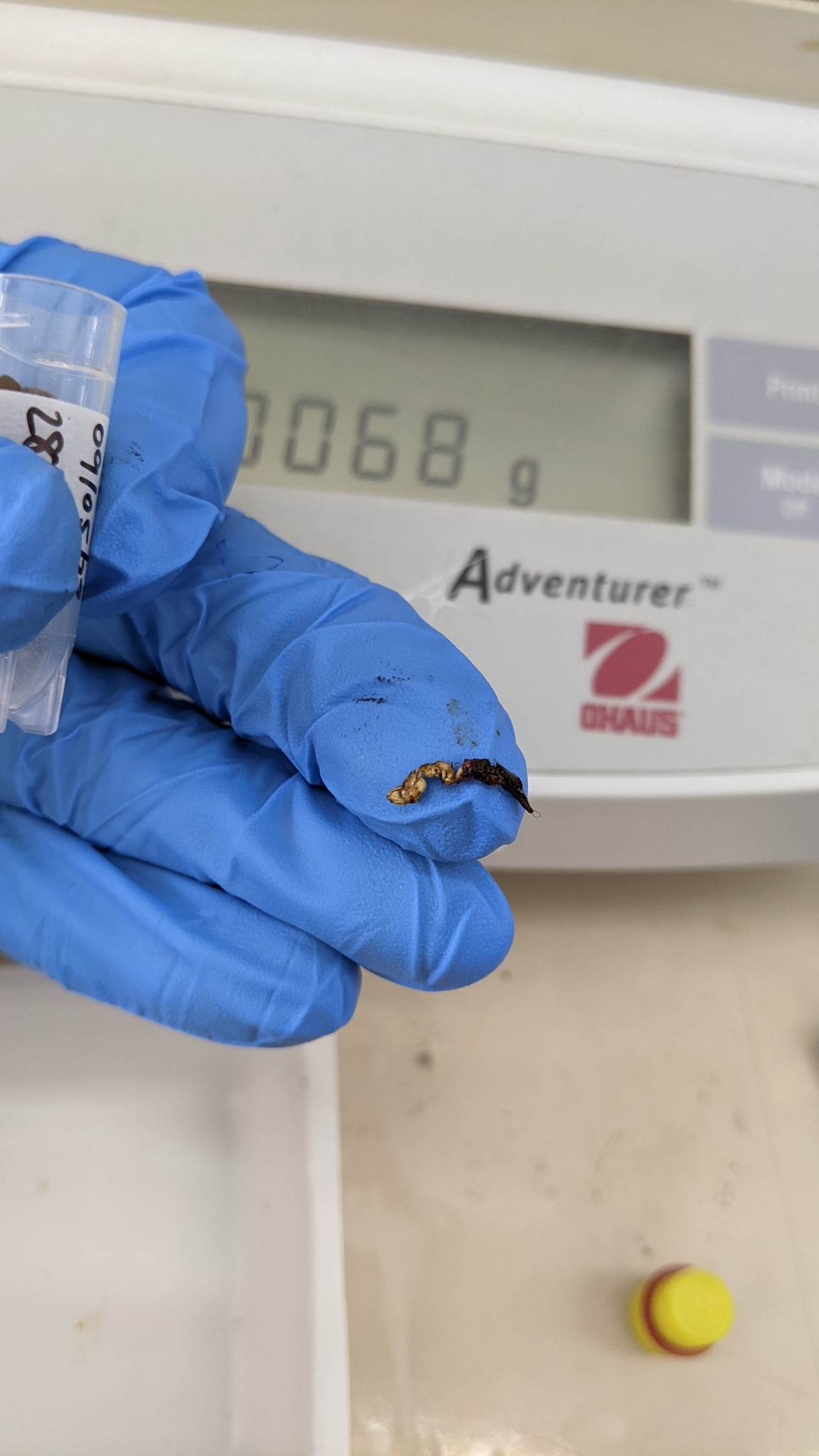

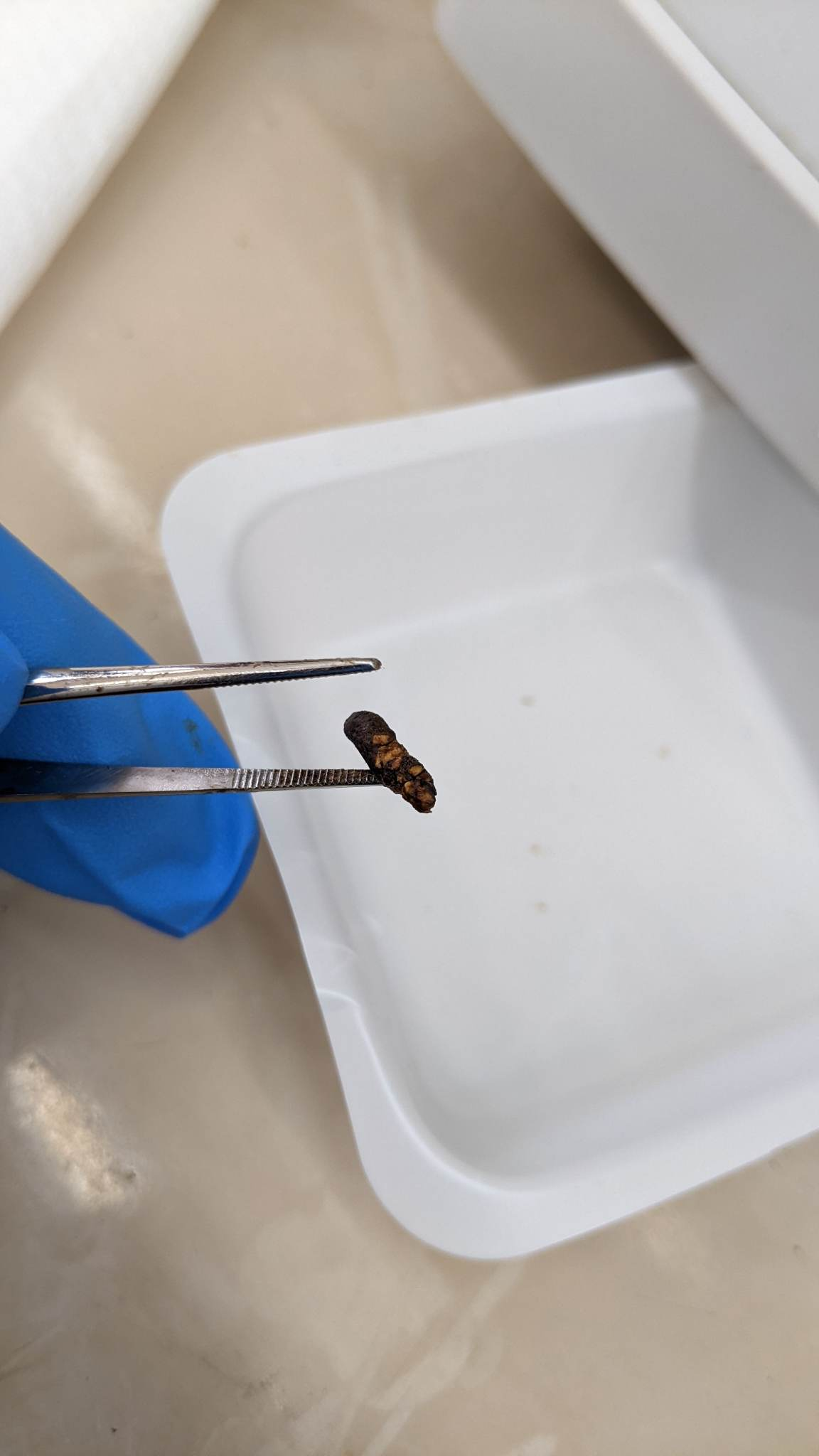
**

a)

b)

c)

**Figure 2:** Fat-tailed dunnart faecal matter showing ingestion of a) MiniFlake sawdust, b) corn cob grid and c) Alpha-dri paper pellets, leading to gastrointestinal compaction.


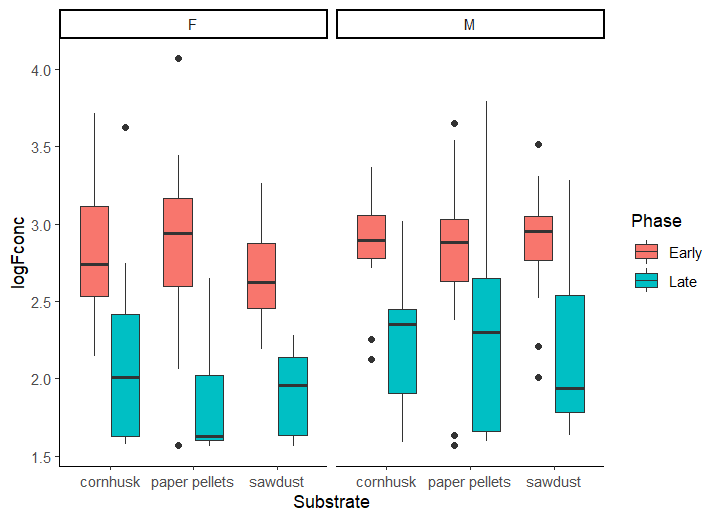


**Figure 3.** Sex differences in response to substrate transition (not significantly different) (p = > 0.005).
